# Neuronal ferroptosis after intracerebral hemorrhage

**DOI:** 10.3389/fmolb.2022.966478

**Published:** 2022-08-05

**Authors:** Siying Ren, Yue Chen, Likun Wang, Guofeng Wu

**Affiliations:** ^1^ Department of Emergency, Affiliated Hospital of Guizhou Medical University, Guiyang, China; ^2^ Graduate School of Guizhou Medical University, Guiyang, China

**Keywords:** intracerebral hemorrhage, secondary brain injury, cell death, ferroptosis, iron overload, oxidative damage, reactive oxygen species

## Abstract

Intracerebral hemorrhage (ICH) is a devastating form of stroke with high rates of morbidity, mortality, and disability. It induces cell death that is responsible for the secondary brain injury (SBI). The underlying mechanism of SBI after ICH is still unclear, and whether it is related to iron overload is worthy to be discussed. Ferroptosis is an iron-dependent non-apoptotic modes of cell death and plays a particularly important role in the occurrence and progression of ICH. Many ICH-induced regulators and signalling pathways of ferroptosis have been reported as promising targets for treating ICH. In this article, we review the definition, characteristics, and inhibition methods of neuronal ferroptosis caused by iron deposition after ICH, and review the biomarkers for ferroptosis.

## 1 Introduction

Intracerebral hemorrhage (ICH) is a subtype of stroke with the highest morbidity, mortality, and disability (van Asch et al., 2010). After ICH occurs, in addition to the primary brain injury caused by the hematoma compressing the surrounding brain tissue, blood components, haemoglobin (Hb), iron, and other neurotoxic substances released by the hematoma also contribute to neuroinflammation and oxidative stress, increase the synthesis of reactive oxygen species (ROS), resulting in secondary brain injury (SBI) (Xi et al., 2006; Wang, 2010). Both the primary and SBI trigger significant cell death and loss of neurological functions. In consideration of the physical compression of the hematoma, many studies have explored the effect of surgical removal of hematoma; however, no significant benefits have been found for patients with ICH (Xi et al., 2014). Similarly, there are limited medical therapy available to alleviate SBI effectively (Bai et al., 2020).

The mechanism of SBI after ICH is complex and remains unclear. Modes of cell death which ICH-induced including necroptosis, pyroptosis, ferroptosis, autophagy, and parthanatos (Zhang et al., 2022). These models of cell death may be associated with SBI after ICH simultaneously. Thus, a better understanding of the modes of cell death in ICH should provide new insights to counter the pathology of ICH. It could result in more effective and targeted neuroprotective or neurorestorative therapeutic strategies (Guo et al., 2020; Zhang et al., 2022).

Studies have shown that excess Hb and iron ions released from hematomas accumulate in the brain parenchyma, causing neurotoxicity and accelerating neurodegeneration (Zecca et al., 2004; Wang et al., 2002). Iron-dependent non-apoptotic cell death, namely, ferroptosis, has been identified as a potential therapeutic target for ICH (Dixon et al., 2012). Neuronal ferroptosis plays key roles in SBI caused by ICH (Xu et al., 2018). Limiting ferroptosis caused by ferrotoxicity and excess accumulation of ROS reduces brain damage and improves the clinical outcomes of patients with ICH (Wang, 2010; Wu L et al., 2012). Therefore, we review the research progress of neuronal ferroptosis, and provide new ideas for treating SBI following ICH.

## 2 Definition and characteristics of ferroptosis

ICH refers to blood entering into the brain parenchyma, ventricle system, or subarachnoid space from a fracturing intracerebral vessel. The mortality rate in ICH patients is 50% approximately, while most of the survivors lose the capability of living independently (Gross et al., 2019). Especially, SBI, which refers to oxidative stress (Yao et al., 2021), inflammation (Xue and Yong, 2020), blood-brain barrier (BBB) hyperpermeability (Keep et al., 2018), and cerebral vasospasm (Athiraman and Zipfel, 2021), further drives brain cell death. Iron is a major product of lysed erythrocytes in hematoma. It can form highly toxic hydroxyl radicals to attack DNA, proteins, and lipid membranes, thereby disrupting cellular functions and causing neuronal death. Iron released from haemoglobin triggers ROS formation, which also induced ferroptosis (Magtanong and Dixon, 2018; Zhang et al., 2022) and is required for the accumulation of lipid peroxides and the execution of ferroptosis (Stockwell et al., 2017). Ferroptosis is iron-dependent non-apoptotic cell death and may play an important role in the development of SBI following ICH.

### 2.1 Definition of ferroptosis

Ferroptosis, a newly identified regulated cell death (RCD) type. More and more researches in this field have revealed the potential roles of ferroptosis in development, immune system regulation, ischemia-reperfusion injury, and tumor suppression (Stockwell et al., 2017). Ferroptosis was first discovered and reported by Dixon et al. in 2012 (Dixon et al., 2012). Ferroptosis is an iron-dependent non-apoptotic form of cell death, which occurs through excessive accumulation of ROS when glutathione (GSH) peroxidase 4 (GPX4), a lipid peroxide reduction system, is dysregulated or relatively insufficient. The Nomenclature Committee on Cell Death (NCCD) defined ferroptosis as “a form of RCD initiated by oxidative perturbations of the intracellular microenvironment that is under constitutive control by GPX4 and can be inhibited by iron chelators and lipophilic antioxidants” (Galluzzi et al., 2018). Iron, lipid, and ROS are three core components of ferroptosis. Metabolic dysregulation of any one of them may influence ferroptotic cell death. Any molecular change or pharmacological intervention that regulates any of these elements may affect the final consequences of ferroptosis (Liu and Gu, 2022).

### 2.2 Characteristics of ferroptosis

Ferroptosis is distinct from apoptosis, necrosis, and autophagy in terms of morphological, biochemical, and genetic properties (Dixon et al., 2012). Electron microscopy has shown that the morphological characteristics of ferroptosis include an obvious reduction in mitochondrial volume, increased density of the mitochondrial double membrane structure, reduced or disappeared cristae formed by the inner membrane, and a near absence of other obvious morphological changes before cell death, particularly the intact nuclei (Yu et al., 2017). The biochemical characteristics of ferroptosis include excessive ROS accumulation, which is dependent on iron ions (Dixon et al., 2012). During the oxidative phosphorylation of mitochondria, cells generate a certain amount of ROS and adenosine triphosphate (ATP). However, a level of ROS that exceeds the antioxidant capacity of cells leads to an enhanced oxidative stress response, which directly or indirectly damages proteins, nucleic acids, lipids, and other macromolecular substances, leading to cell damage or cell death (Abrams et al., 2016). The genetic characteristics of ferroptosis are regulated by a unique set of genes, mainly including ribosomal protein L8 (*RPL8*), ATP synthase F_0_ complex subunit C3 (*ATP5G3*), iron response element binding protein 2 (*IREB2*), citrate synthase (*CS*), tetratricopeptide repeat domain 35 (*TTC35*), and acyl-CoA synthetase family member 2 (*ACSF2*) genes (Dixon et al., 2012).

### 2.3 Autophagy promotes ferroptosis

Ferroptosis is caused by dysregulated cell metabolism, including iron, lipid, amino acids, and ROS metabolism (Liu and Gu, 2022). Gao et al. showed that autophagy promotes ferroptosis by regulating intracellular iron homeostasis and ROS synthesis (Gao et al., 2016). *In vitro* experiments showed that the application of Erastin, a synthetic small-molecule compound, which induces ferroptosis and activates autophagy, led to intracellular ferritin degradation to further increase the level of intracellular iron ions through autophagy, resulting in rapid accumulation of intracellular ROS, which promote ferroptosis. Hou et al. also demonstrated that the activation of autophagy further promoted ferroptosis by degrading ferritin in tumor cells (Hou et al., 2016).

## 3 Discovery of neuronal ferroptosis after ICH

Li et al. used a collagenase-induced ICH mouse model to observe morphological changes and showed that the proportion of shrunken mitochondria in the cytoplasm and axon of neurons around the hematoma increased, which strongly confirmed the occurrence of ferroptosis and was the earliest report of neuronal ferroptosis after ICH. They also applied ferrostatin-1 (Fer-1),a specific ferroptosis inhibitor after acute ICH and showed that Fer-1 improved the neurological functions of the mice (Li et al., 2017a). In the same year, Zille et al. showed that the ICH model pretreated with Hb had increased levels of phosphorylated extracellular regulated protein kinases (ERK1/2) (Zille et al., 2017). As ERK1/2 is an important signaling molecule in the RAS-RAF-MEK pathway in the process of ferroptosis induced by the small-molecule Erastin, the finding of Zille et al. further prove from a molecular viewpoint that neurons undergo ferroptosis after ICH. In 2018, Li et al. used transmission electron microscopy (TEM) to monitor the ultra-micromorphological changes of neurons in a collagenase-induced ICH animal model and showed that on the third and sixth day after ICH, both the soma and axon of neurons had an increased proportion of shrunken mitochondria (Li et al., 2018), providing sufficient evidence for the occurrence of neuronal ferroptosis after ICH. Recently, an increasing number of researchers have investigated neuronal ferroptosis after ICH, with the aim to identify new directions and targets for treating SBI after ICH.

## 4 Biomarkers for neuronal ferroptosis after ICH

Observations of morphological changes by TEM or pharmacological and molecular characteristics are the most common approaches to identify neuronal ferroptosis. Although these are relatively complicated processes, the identification of biomarkers of ferroptosis will bring great convenience to the determination of ferroptosis in the future. Li et al. used Hb to stimulate hippocampal tissue slice culture *in vitro* and showed high expression of prostaglandin endoperoxide synthase 2 (PTGS2), but no significant changes in the mRNA levels of *IREB2*, *CS*, *RPL8*, and *ATP5G3* (Li et al., 2017b). PTGS2 encodes cyclooxygenase-2 (COX-2), which is a key enzyme in prostaglandin biosynthesis. Studies have confirmed that ferroptosis is regulated by PTGS2, a unique and potentially direct downstream gene that induces ferroptosis. COX-2 is highly expressed after ICH and its inhibition alleviates the SBI caused by ICH, suggesting that COX-2 may be a biomarker for neuronal ferroptosis after ICH. However, existing research is limited to the Hb-induced ICH mouse model, and further research is needed to confirm the sensitivity, representativeness, and reproducibility of using COX-2 as a biomarker for neuronal ferroptosis after ICH (Chen et al., 2019).

## 5 Mechanism of neuronal ferroptosis after ICH

### 5.1 Intracellular iron overload

**FIGURE 1:**
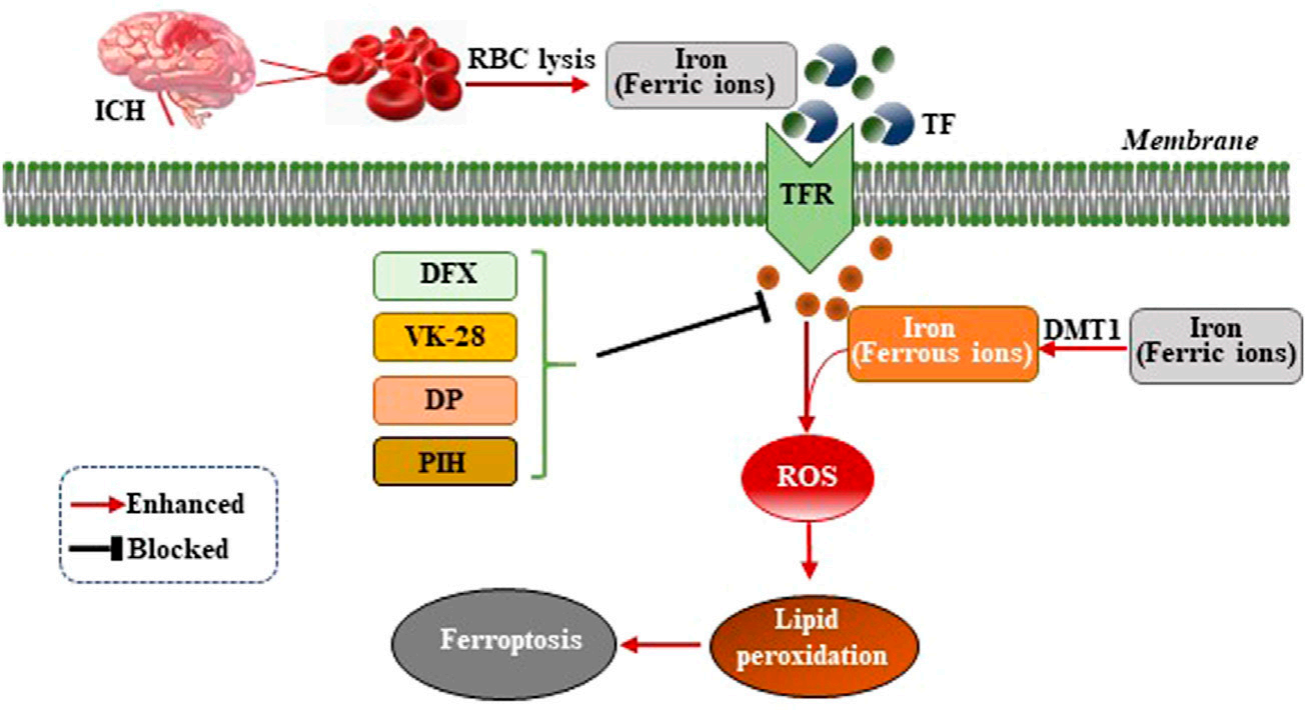
Mechanism of ferroptosis following ICH by intracellular iron overload. After ICH occurs, the hematoma releases iron ions from RBC lysis, the concentration of iron ions increase around neurons and combine with transferrin (TF) to transport iron ions into cells through TF receptor (TFR)-mediated effects. Ferric ions are reduced to ferrous ions by divalent metal transporter 1 (DMT1) and accumulate in neurons, where ferrous ions induce excessive generation of lethal ROS and lipid peroxides, therefore, resulting in ferroptosis. Iron chelators (DFX, VK-28, DP, and PIH) form a chelating ferric amine with iron ions to prevent iron ions from donating electrons to oxygen to form ROS.

Iron ions are essential for the occurrence of ferroptosis. However, the specific mechanism by which iron ions promote ferroptosis is not yet fully understood (Cao and Dixon, 2016). After ICH, the excess ferric ions from the red blood cells (RBC) lysis combine with transferrin (TF) in the serum to transport iron ions into cells through receptor-mediated effects (Andrews, 2000). Ferric ions are reduced to ferrous ions by divalent metal transporter 1 (DMT1) and accumulate in neurons, where ferrous ions induce excessive generation of lethal ROS and lipid peroxides (Figure 1). A previous study of the ICH mouse model showed that two iron chelators, deferoxamine (DFX) and VK-28 (5-[4-(2-hydroxyethyl) piperazine- 1-ylmethyl]-quinoline-8-ol), reduced neuronal death, ROS synthesis, accumulation of iron ions around the hematoma, activation of microglia, and improved the neurological function of the mouse model (Li et al., 2017a). Wu et al. demonstrated that fat-soluble iron chelator, 2,2′-dipyridy (DP), reduced iron deposition around the hematoma and ROS synthesis, improving the neuronal function and reducing the neuronal death of the ICH mouse model (Wu L et al., 2012). Pyridoxal isonicotinoyl hydrazine (PIH), a lipophilic iron-chelating agent, has been reported to reduce excess iron-induced cytotoxicity following ICH, which was associated with mitigation of inflammation and ferroptosis (Zhang H et al., 2021). The effect of iron ion chelators on the improvement of neuronal function in mice after ICH indicated that the occurrence of ferroptosis was inseparable from the effect of iron ions (Figure 1). Nevertheless, the specific mechanism still needs further exploration.

### 5.2 Inhibiting the cystine/glutamate antiporter

**FIGURE 2:**
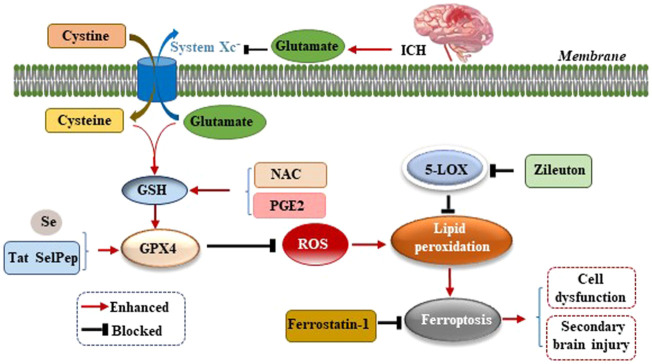
Mechanism of ferroptosis following ICH by inhibiting cystine/glutamate antiporter (System Xc^−^). After ICH occurs, excessive glutamate (Glu) around neurons inhibits the activity of System Xc^−^ and the transfer of cysteine (Cys), leading to reduction of glutathione (GSH) synthesis, which reduces the activity of glutathione peroxidase 4 (GPX4), resulting in excessive reactive oxygen species (ROS) and lipid peroxide levels which cannot be scavenged, thereby leading to neuronal ferroptosis, cell dysfunction, and secondary brain injury (SBI) caused by ICH.

Studies have confirmed an increased level of glutamate (Glu) in the brain tissue surrounding the hematoma after ICH in mice, rabbits, and patients with ICH (Li et al., 2017b; Wu et al., 2013; Castillo et al., 2002). The addition of Glu to the culture medium of HT22 hippocampal neurons led to a significant increase in cell death, and the application of the 5-lipoxygenase (5-LOX) inhibitor Zileuton suppressed 5-LOX to reduce lipid peroxide production, which inhibits ferroptosis and thereby protects neurons (Liu et al., 2015). Li et al. observed an increase in the level of Glu around the hematoma of the ICH mouse model and used the glutaminase inhibitor 968 to inhibit the decomposition of glutamine into Glu to significantly reduce the number of degenerated neurons around the hematoma, suggesting that the breakdown of glutamine led to neuronal ferroptosis after ICH *in vivo* (Li et al., 2017a). The reason for this finding may be that excessive Glu around neurons inhibits the activity of cystine/glutamate antiporter (System Xc^−^) and the transfer of cysteine (Cys). As Cys is the raw material for GSH synthesis, the reduction of Cys leads to a further reduction of GSH synthesis, which reduces the activity of GPX4 (Figure 2). This, results in excessive ROS and lipid peroxide levels which cannot be scavenged, thereby leading to neuronal ferroptosis and aggravating the dysfunction, cerebral edema, oxidative stress, BBB damage, and inflammatory response caused by ICH (Gao et al., 2015). Up-regulating the expression of GPX4 in ICH model can inhibit ferroptosis and treat ICH (Peng et al., 2022).

### 5.3 P53 in ferroptosis regulation

The tumor suppressor p53 as a master regulator of ferroptosis, has been among the most extensively studied genes since its discovery in 1979. A major function of p53 is mediating cellular and systematic metabolism. Interestingly, p53 is tightly associated with all key metabolic pathways involved in ferroptosis. Unlike apoptotic cell death, activation of p53 solely is not sufficient to induce ferroptosis directly; instead, through its metabolic targets, p53 is able to modulate the ferroptosis response in the presence of ferroptosis inducers such as GPX4 inhibitors or high levels of ROS. More and more studies to this day have been revealed that p53 is a key regulator of both canonical and non-canonical ferroptosis pathways via a variety of mechanisms. In most cases, p53 promotes ferroptosis. However, under a certain context, p53 can inhibit ferroptosis (Liu and Gu, 2022).

#### 5.3.1 P53 modulates GPX4-dependent ferroptosis pathways

**FIGURE 3:**
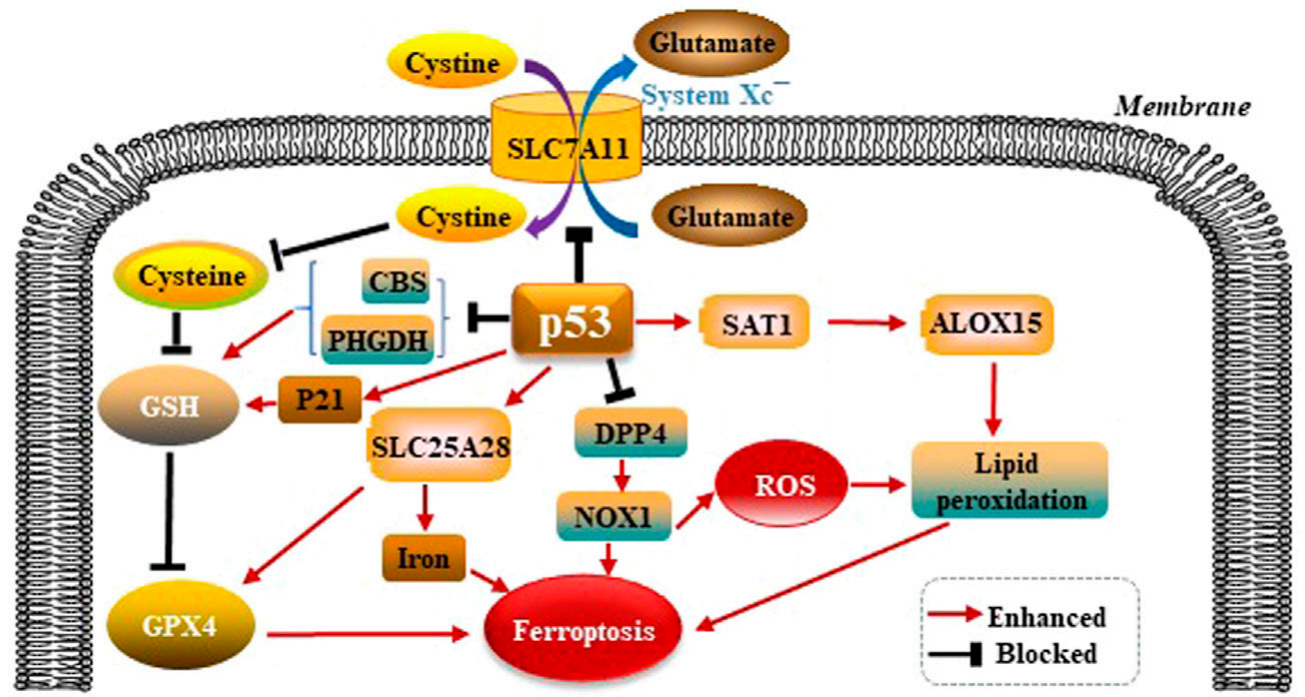
p53 modulates GPX4-dependent ferroptosis pathways. p53 suppresses the transcription of SLC7A11, which is a core subunit of the System Xc^−^. SLC7A11 mediates cellular uptake of extracellular cystine in exchange for intracellular Glu, reduces downstream GSH and GPX4 biosynthesis, thus leading to ferroptosis. p53 also promotes ferroptosis through regulating other metabolic pathways. p53 can upregulate ALOX15 via SAT1-mediated, leads to lipid peroxidation and ferroptosis. p53 enhances the activity of SLC25A28, causes abnormal accumulation of redox-active iron and promotes ferroptosis. p53 also can suppress PHGDH and CBS respectively, limiting GSH production. p53 inhibits DPP4 by binding NOX1, boosting the production of ROS, and resulting in lipid peroxidation and ferroptosis. p21 is a target gene of p53. p21 suppresses GSH synthesis, and promotes ferroptosis.

p53 was shown to promote ferroptosis via its capacity to inhibit the import of cystine into target cells. Mechanistically, p53 was found to suppress the transcription of SLC7A11 solute carrier family 7 member 11 (SLC7A11), which is a core subunit of the System Xc^−^. The activity of System Xc^−^ is mainly determined by SLC7A11. SLC7A11 was first identified as a direct target gene suppressed by p53 (Jiang et al., 2015). SLC7A11 mediates cellular uptake of extracellular cystine in exchange for intracellular Glu. Interference of cystine absorption reduces downstream GSH bio- synthesis and thus decreases GPX4’s ability to antagonize ferroptosis. p53 also promotes ferroptosis through regulating other metabolic pathways. Spermidine/spermine N1-acetyltrans- ferase 1 (SAT1) is a rate-limiting enzyme in polyamine catabolism. p53 can transactivate SAT1. SAT1 induction leads to lipid peroxidation and ferroptosis. This effect is due to arachidonate-15-lipoxygenase (ALOX15) upregulation after SAT1 induction. Therefore, the p53/SAT1/ALOX15 axis partially contributes to p53-mediated ferroptosis. p53 enhances the activity of SLC25A28 solute carrier family 25 member 28 (SLC25A28), a protein coding gene that causes abnormal accumulation of redox-active iron and promotes ferroptosis (Zhang et al., 2020). p53 can inhibit the serine synthesis pathway and transsulfuration pathway by suppressing phosphoglycerate dehydrogenase (PHGDH) and cystathionine β-synthase (CBS) respectively, limiting GSH production (Ou et al., 2015; Wang et al., 2019). In summary, p53 promotes ferroptosis through its multipotent roles in regulating cellular metabolism, particularly lipid, iron, ROS, and amino acid metabolism (Liu and Gu, 2021). Mechanistically, dipeptidyl peptidase 4 (DPP4) promotes ferroptosis in p53-deficient cells by binding nicotinamide adenine dinucleotide phosphate (NADPH) oxidase 1 (NOX1) and boosting the production of ROS resulting in lipid peroxidation and ferroptosis (Gao et al., 2019). p21 is a target gene of p53 (Hong et al., 2010), and p21 induction redistributes the serine usage from nucleotide biogenesis to GSH synthesis, and GSH is an inhibitor of ROS and ferroptosis (Tarangelo et al., 2018; Mattocks et al., 2013). p53 modulates GPX4-dependent ferroptosis pathways as is shown in the Figure 3.

#### 5.3.2 P53 modulates GPX4-independent ferroptosis pathways

**FIGURE 4:**
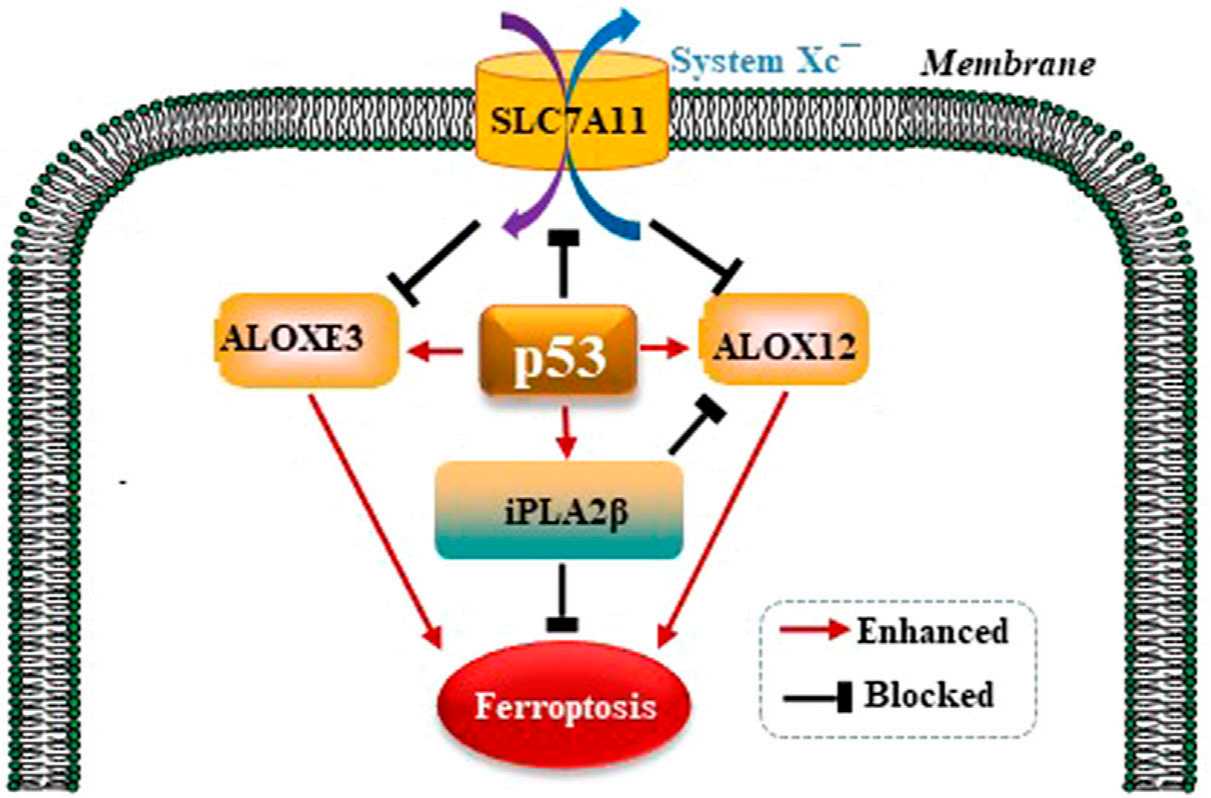
p53 modulates GPX4-independent ferroptosis pathways. p53/ SLC7A11/ALOX12 and p53/SLC7A11/ALOXE3 axes can both collaboratively and independently in modulating ferroptosis in a GPX4-independent manner. iPLA2β-mediated detoxification of peroxidated membrane lipids is adequate to inhibit p53/ALOX12-driven ferroptosis also in a GPX4-independent pattern.

It is well established that ferroptosis is controlled by GPX4 primarily. Amazingly, Chu et al. observed that p53 modulates ferroptosis without apparent effects on GPX4. By screening the ALOX arachidonate lipoxygenase family to identify potential contributors to p53-mediated ferroptosis, founding that ALOX12 is a critical candidate (Chu et al., 2019). p53 promotes the activity of ALOX12 via inhibiting SLC7A11 which binds and sequesters ALOX12 from its substrate, polyunsaturated fatty acid (PUFA), including those esterified in membranes. When p53 downregulates SLC7A11, ALOX12 is released and subsequently oxidizes membrane PUFAs and initiates ferroptosis. Therefore, the p53/SLC7A11/ALOX12 axis is independent of the reduce in GSH and GPX4 activity, and is a mechanism underlying different than the p53/ SLC7A11/GPX4 pathway. In a recent study, Yang et al. reported that another ALOX family member, ALOXE3, acts in a similar way to ALOX12 in generating ferroptosis (Yang et al., 2021). SLC7A11 also binds and sequesters ALOXE3 from its substrate. p53/SLC7A11/ ALOXE3- mediated ferroptosis is independent of long-chain acyl-CoA synthetases 4 (ACSL4). In consideration of that ALOXE3 is downstream of ALOX12, the p53/ SLC7A11/ALOX12 and p53/SLC7A11 /ALOXE3 axes can both collaboratively and independently in modulating ferroptosis. Phospholipase A2 group VI (iPLA2β) is a calcium-independent phospholipase that gaps acyl tails from the glycerol backbone of lipids and releases oxidized fatty acids, which can be further detoxified by the antioxidants in the cytoplasm (Malley et al., 2018). iPLA2β-mediated detoxification of peroxidated membrane lipids is adequate to inhibit p53/ALOX12-driven ferroptosis in a GPX4-independent pattern (Liu and Gu, 2022). P53 modulates GPX4-independent ferroptosis pathways as is shown in the Figure 4.

#### 5.3.3 p53 modulates ferroptosis in ICH

**FIGURE 5:**
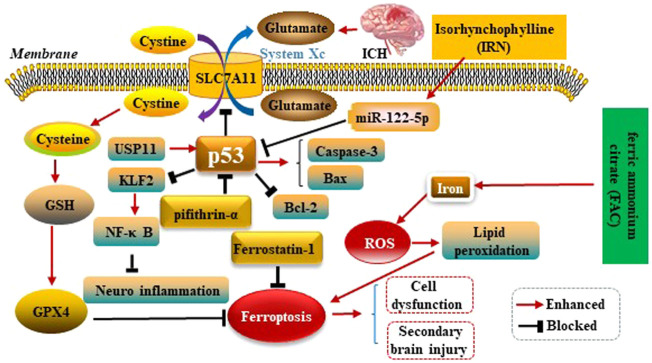
p53 modulates ferroptosis in ICH. p53 inhibits SLC7A11 resulting in dysfunction of the cystine/glutamate antiporter (System Xc^−^) after ICH, leading to decreased synthesis of glutathione (GSH) and activity of glutathione peroxidase 4 (GPX4). p53 could increase Caspase-3 as well as Bax expressions, and decrease Bcl-2 expression conversely. Therefore, P53 plays key roles in SBI caused by ICH. IRN suppressed the p53 expression to anti-ferroptosis via miR-122–5p/p53/SLC7A11 pathway. Ubiquitin-specific protease 11(USP11) stabilized p53 by deubiquitination and p53 targeted Krüppel like factor 2 (KLF2) promoter to repress KLF2 transcription, thereby activating the nuclear factor-kappa B (NF-κB) pathway to anti-ferroptosis. Ferric ammonium citrate (FAC) could be a source of iron to induce iron overload, thus leading to ferroptosis.

Kuang et al.observed ferroptosis characteristics in the cerebral cortex of rats with subarachnoid haemorrhage (SAH) after 24 h, and could be alleviated by Fer-1 treatment. Fer-1 could increase SLC7A11 and GPX4. Similarly, BBB impairment, brain edema, behavioral deficits and neuronal damage were relieved by inhibiting ferroptosis. Moreover, the p53 inhibitor Pifithrin-α could block cortical SAH-induced ferroptosis. These results indicated that ferroptosis aggravated SBI after SAH was partly dependent on p53, and inhibiting ferroptosis might be an effective therapeutic target for SBI (Kuang et al., 2021). Zhang et al. used ICH rats model to explore the mechanism of Ubiquitin-specific protease 11(USP11) regulating neuroinflammation in ICH. It was showed in microglial cells that USP11 stabilized p53 by deubiquitination and p53 targeted Krüppel like factor 2(KLF2) promoter to repress KLF2 transcription, thereby activating the nuclear factor-kappa B (NF-κB) pathway. Further, rescue experiments were conducted *in vivo* to make sure the function of USP11/p53/KLF2/NF-κB axis in ICH-induced inflammation, which confirmed that USP11 silencing inhibited the release of pro-inflammatory cytokines following ICH by downregulating p53, thus protecting against neurological deficit. (Zhang X et al., 2021). Xu et al. first reported p53 could increase apoptosis-associated protein cysteine aspartate protease-3 (Caspase-3) as well as Bcl-2 associated X protein (Bax) expressions, and decrease B-celllymphoma-2 (Bcl-2) expression conversely. Therefore, p53 plays key roles in SBI caused by ICH (Xu et al., 2018). Isorhynchophylline (IRN), a component of traditional Chinese herb Uncaria rhynchophylla, possesses strong antioxidant activity. A present study showed IRN exhibited neuroprotective effects *in vivo* and *in vitro* by inhibiting ferroptosis. In particular, IRN suppressed the p53 expression to promote the transcription of SLC7A11 by upregulating the miR-122–5p expression, thus exerting its anti-ferroptosis activity. The fndings reveal that IRN protects neurocyte from ferric ammonium citrate (FAC) -induced ferroptosis via miR-122–5p/ p53/SLC7A11 pathway, which may provide a potential therapeutic mechanism for ICH (Zhao et al., 2021). p53 modulates ferroptosis in ICH as is shown in the Figure 5.

In conclusion, how can p53 enhance or block ferroptosis are developing, and many problems remain unknown. Emerging research results have increasingly advanced our knowledge of p53. p53 and ferroptosis fields need much more research to obtain more achievements.

## 6 Inhibition of neuronal ferroptosis after ICH

### 6.1 Ferroptosis inhibitor (Fer-1)

Fer-1 is a specific ferroptosis inhibitor. Dixon et al. showed that Fer-1 inhibited Hb-induced cell death in hippocampal slice culture, confirming that Fer-1 reduced iron ion-induced cell death and the release of lactate dehydrogenase (LDH) induced by Hb and ferrous ions. Additionally, the content of ROS in the cultured hippocampal slices in the experimental group with Fer-1 intervention was significantly lower than that in the control group (Dixon et al., 2012). Zhang et al. (Zhang et al., 2018) also confirmed that Fer-1 significantly reduced the levels of interleukin-1 beta (IL-1β) and tumor necrosis factor-alpha (TNF-α) in the serum and cerebrospinal fluid, which in turn alleviated the inflammatory response of rats after ICH, reduced albumin extravasation and BBB damage, and alleviated SBI after ICH. Generally, the *in vivo* and *in vitro* experiments have confirmed that the application of Fer-1 inhibits the occurrence of ferroptosis, thereby alleviating the SBI after ICH and providing a theoretical basis for further clinical application of Fer-1. Fer-1 is listed in Supplementary Table S1 which summarize the potential inhibition methods of ferroptosis following ICH.

### 6.2 Iron chelator (DFX, VK-28, DP and PIH)

The iron chelator DFX reduces brain edema, neurological deficit, and brain atrophy in rats after ICH (Okauchi et al., 2010). The sustained release of iron ions from the hematoma after ICH activates local microglia and causes secondary brain damage. DFX forms a chelating ferric amine with iron ions in the brain tissue around the hematoma to prevent iron ions from donating electrons to oxygen to form ROS and inhibits the hyperactivation of microglia (Li et al., 2017b; Dixon and Stockwell, 2014), which is the most important mechanism of neuronal ferroptosis. Another study showed that the application of DFX or VK-28 6 h after ICH in mice effectively reduced ROS synthesis by approximately 50%, thereby reducing neuronal cell death after ICH and improving secondary neuronal damage. Compared to DFX, the iron chelator VK-28 had a greater advantage of penetrating the intact BBB, while DFX failed to do so. Thus, VK-28 may act in the brain at a relatively low concentration, which is more suitable for clinical application (Li et al., 2017a). Generally, iron chelators further inhibit the occurrence of ferroptosis by forming chelating ferric amines with iron ions in the brain tissue around hematoma. VK-28 is more effective and safer than DFX in inhibiting ferroptosis. Additionally, a previous study showed that fat-soluble iron chelator, 2,2′-dipyridy (DP), reduced iron deposition around the hematoma and ROS synthesis, improving the neuronal function and reducing the neuronal death of the ICH mouse model (Wu H et al., 2012).

Pyridoxal isonicotinoyl hydrazine (PIH), a lipophilic iron-chelating agent, has been reported to reduce excess iron-induced cytotoxicity after ICH, which was associated with mitigation of inflammation and ferroptosis (Zhang H et al., 2021). Iron chelators (DFX, VK-28, DP and PIH) are listed in Supplementary Table S1 which summarize the potential inhibition methods of ferroptosis following ICH.

### 6.3 DMT1 inhibitor (ebselen)

DMT1 is involved in iron ionization in cells and is the only protein that transports iron ions into cells (Gao et al., 2015). After ICH, the expression of DMT1 is significantly increased, and ferric ions are reduced to ferrous irons under the action of DMT1. Ferrous ions induce excessive ROS and lipid peroxide production and are important factors causing neuronal ferroptosis (Xie et al., 2012). Pretreatment with Ebselen, a DMT1 inhibitor, significantly reduces the iron ion transport activity of DMT1 and suppresses ROS synthesis (Wang et al., 2016). A previous study from China showed that Ebselen further reduced neuronal ferroptosis after subarachnoid hemorrhage in rats by inhibiting DMT1 (Zhang et al., 2017). Hence, it is necessary to further strengthen the research on Ebselen in the context of ICH. Ebselen is listed in Supplementary Table S1 which summarize the potential inhibition methods of ferroptosis following ICH.

### 6.4 Flavanol compound (epicatechin)

Epicatechin (EC) is a flavanol compound present in natural plants (e.g., fruits, vegetables, and green tea). EC activates NF-E2-related factor (Nrf2) signaling to scavenge oxidants and free radicals. Existing reports have shown that the application of EC reduced the volume of brain damage and brain edema, improved neurological function and prognosis, and significantly reduced *IREB2* mRNA expression in the early stage of ICH in mice. *IREB2* is involved in encoding iron-ion regulators and inhibits neuronal ferroptosis by further regulating the intracellular iron-ion metabolism (Chang et al., 2014). After ICH, the neuronal cell viability of *IREB2*-knockout mice was higher than that of the mice in the *IREB2-*non-knockout group, showing a relatively strong resistance to Hb toxicity (Regan et al., 2008). Importantly, EC passes through the BBB and is convenient to use (Wu L et al., 2012). Hence, EC has promising clinical research value for inhibiting neuronal ferroptosis and SBI after ICH. EC is listed in Supplementary Table S1 which summarize the potential inhibition methods of ferroptosis following ICH.

### 6.5 Combined application of n-acetyl-l-cys and prostaglandin E2

NAC is a Cys precursor approved by the Food and Drug Administration of the United States for clinical use for treating paracetamol-induced liver failure (Green et al., 2013). Much preclinical data are available to support the use of NAC in acute and chronic neurological and psychiatric disorders (Ansari et al., 2019; De Rosa et al., 2015; Monti et al., 2016). Cys, as a component of the cellular reducing agent GSH, is necessary to maintain cellular redox homeostasis; hence, Cys deficiency further depletes intracellular GSH, thereby increasing ROS synthesis and inducing ferroptosis. Under these circumstances, supplementation with another biosynthetic precursor of GSH, i.e., NAC, can effectively prevent cells from undergoing ferroptosis (Liu et al., 2015). A study by Zille et al. on ICH in male mice showed that NAC inhibited neuronal ferroptosis *in vitro* (Zille et al., 2017). Moreover, Karuppagounder et al. confirmed that NAC prevented heme oxygenase-1 induction by neutralizing toxic lipids produced by arachidonic acid-dependent 5- LOX 7 days after ICH, resulting in reduced neuronal cell death after ICH and improved functional recovery. The application of PGE2 combined with NAC may have a synergistic effect and reduce the NAC administration dosage required for the protection and functional recovery of brain tissue. Nevertheless, the application of NAC may cause serious adverse reactions. Thus, further research should focus on controlling NAC dosage and its adverse reactions (Karuppagounder et al., 2018). Combined NAC and PGE2 are listed in Supplementary Table S1 which summarize the potential inhibition methods of ferroptosis following ICH.

### 6.6 Supplementation with selenium

Ingold et al. reported that Se effectively inhibited GPX4-dependent ferroptosis and synergistically activated transcription factor activator protein 2C (TFAP2C) and transcription factor Sp1 (TSFP1), which further activated GPX4 transcription (Ingold et al., 2018). Another study confirmed that an intraventricular injection of sodium selenite promoted the expression of TSFP1 and GPX4 in neurons of the ICH mouse model, significantly reducing the neuronal death rate and promoting the recovery of nerve function in the mice (Alim et al., 2019). However, intraventricular injection requires the insertion of a catheter or needle into the brain tissue, which is invasive and carries the risk of developing intracranial infection and exacerbating the disease (Alim et al., 2019; Ding et al., 2015). Additionally, Se has a narrow therapeutic window and causes neurotoxicity if the dose is not titrated within an appropriate range (Fam et al., 2017). To overcome this problem, Alim et al. developed a Se-containing Cys polypeptide, Tat SelPep, to not only penetrate the BBB in a broad therapeutic window, but also to induce high GPX4 expression in various organs including the brain, heart, and liver. Se and its related new drugs provide additional potential treatment options for diseases related to GPX4 deficiency, including ICH. Se or Tat SelPep is listed in Supplementary Table S1 which summarize the potential inhibition methods of ferroptosis following ICH.

## 7 Summary and outlook

ICH is the stroke subtype with the highest mortality rate of acute cerebrovascular disease, accounting for 15–20% of all strokes, with no effective treatments currently available (Feigin et al., 2009; Keep et al., 2012). Neuronal cell death after ICH includes necroptosis, pyroptosis, ferroptosis, autophagy, and parthanatos (Zhang et al., 2022). Ferroptosis may play an important role in the development of SBI after ICH. Recently, research on ferroptosis has become popular, and new mechanisms of ferroptosis and its potential clinical functions are still emerging, which are of great significance for exploring the therapeutic directions and intervention targets after ICH. This review article presents the definition of ferroptosis, the discovery of neuronal ferroptosis after ICH, the mechanism of ferroptosis, and the ways in which ferroptosis can be suppressed. Generally, the occurrence of ferroptosis is closely related to the content of intracellular iron ions, GSH, lipid peroxidases. Fer-1, iron-ion chelators, Ebselen, EC, NAC, PGE2, Se, and others, which may suppress ferroptosis after ICH, providing a new direction for the clinical treatment of ICH. However, more in-depth studies are necessary to determine how to best apply the above substances in clinical practice and reduce their related adverse reactions.

## Glossary

ATP: adenosine triphosphate
ATP5G3: ATP synthase F0 complex subunit C3
ACSF2: acyl-CoA synthetase family member 2
ALOX15: arachidonate-15-lipoxygenase
BBB: blood-brain barrier
Bax: Bcl-2 associated X protein
Bcl-2: B-celllymphoma-2
Caspase-3: Cysteine aspartate protease-3
CS: citrate synthase
COX-2: cyclooxygenase-2
Cys: cysteine
CBS: cystathionine β-synthase
DMT1: divalent metal transporter 1
DFX: deferoxamine
DP: 2,2′-dipyridy
DPP4: dipeptidyl peptidase 4
EC: Epicatechin
ERK1/2: extracellular regulated protein kinases
Fer-1: ferrostatin-1
FXDR: ferredoxin reductase
FAC: ferric ammonium citrate
Glu: glutamate
GSH: glutathione
GPX4: glutathione peroxidase 4
HB: hemoglobin
IREB2: Iron response element binding protein 2
ICH: intracerebral haemorrhage
IL-1β: interleukin-1 beta
iPLA2β: Phospholipase A2 group VI
IRN: isorhynchophylline
KLF2: Krüppel like factor 2
LDH: dehydrogenase
5-LOX: 5-lipoxygenase
Nrf2: NF-E2-related factor
NAC: N-Acetyl-L-Cys
NOX1: NADPH oxidase 1
NCCD: The Nomenclature Committee on Cell Death
NADPH: nicotinamide adenine dinucleotide phosphate
NF-κB: nuclear factor-kappa B
PTGS2: prostaglandin endoperoxide synthase 2
PGE2: prostaglandin E2
PIH: pyridoxal isonicotinoyl hydrazine
PHGDH: phosphoglycerate dehydrogenase phosphoglycerate dehydrogenase
PUFA: polyunsaturated fatty acid
ROS: reactive oxygen species
RPL8: ribosomal protein L8
RCD: regulated cell death
SBI: secondary brain injury
SLC7A11: solute carrier family 7 member 11
SLC25A28: olute carrier family 25 member 28
System: Xc- cystine/glutamate antiporter
SAT1: spermidine/spermine N1- acetyltransferase 1
Se: selenium
SAH: subarachnoid haemorrhage
TF: transferrin
TFR: transferrin receptor
TTC35: tetratricopeptide repeat domain 35
TEM: transmission electron microscopy
TNF-α: tumor necrosis factor-alpha
TSFP1: transcription factor Sp1
TFAP2C: transcription factor activator protein 2C
Tat SelPep: Se-containing Cys polypeptide
USP11: Ubiquitin-specific protease 11
