# Supplementary material for: Neuronal ferroptosis after intracerebral hemorrhage
Source: Front Mol Biosci. 2022 Aug 5;9:966478. doi: 10.3389/fmolb.2022.966478 (PMC9388724; doi:10.3389/fmolb.2022.966478)
Supplement: Supplementary file 1 [file Table1.DOCX]

**TABLE 1** The potential inhibition methods of ferroptosis after ICH.

| **Molecule** | **Targets** | **Mechanisms** | **Effects** | **Reference(s)** |
| --- | --- | --- | --- | --- |
| Fer-1 | SLC7A11;GPX4 | Promote System Xc^-^; Increase GPX4 | Anti-ferroptosis; Anti-SBI | Dixon et al.,2012; Zhang et al.,2018 |
| DFX | Iron ions | Inhibit iron deposition; Inhibit ROS; Inhibit the hyperactivation of microglia | Anti-ferroptosis; Anti-SBI | Okauchi et al.,2010; Li et al.,2017; Dixon et al.,2014 |
| VK-28 | Iron ions | Inhibit iron deposition; Inhibit ROS | Anti-ferroptosis; Anti-SBI | Li et al.,2017 |
| DP | Iron ions | Inhibit iron deposition; Inhibit ROS | Anti-ferroptosis; Anti-SBI | Wu et al.,2012 |
| PIH | Iron ions | Inhibit iron deposition; Inhibit ROS | Anti-ferroptosis;Anti-cytotoxicity; Anti-SBI | Zhang et al.,2021 |
| Ebselen | DMT1 | Ferric ions are reduced to ferrous ions by DMT1 | Anti-ferroptosis;Anti-ROS;  Anti-Lipidperoxidation | Gao et al., 2015; Xie et al., 2012; Wang et al.,2015; Zhang et al., 2017 |
| EC | Nrf2 pathways; IREB2 | Scavenge oxidants and free radicals; Encoding iron-ion regulators | Anti-ferroptosis;Anti-ROS;  Anti-SBI | Chang et al.,2014; Regan et al.,2008; Wu et al.,2012 |
| NAC+PGE2 | GSH; ALOX5 | Maintain cellular redox homeostasis | Anti-ferroptosis;Anti-ROS | Green et al.,2013;Ansari et al.,2019; De Rosa et al.,2015; Monti et al., 2016; Liu et al.,2015; Karuppagounder et al.,2018 |
| Se  (Tat SelPep) | TFAP2C; TSFP1 | Activated GPX4 transcription | Anti-ferroptosis | Ingold et al.,2018; Alim et al.,2019; |
| Erastin | Ferritin; Iron ions | Promote ROS | Promote ferroptosis | Hou et al.,2016; Zille et al.,2017 |
| PTGS2 | COX-2 | PTGS2 encodes COX-2 | Promote ferroptosis | Li et al.,2017; Chen et al.,2019 |
| Zileuton | 5-LOX | Reduce lipid peroxide production | Anti-ferroptosis | Liu et al.,2015 |
| Pifithrin-ɑ | p53 | Inhibit p53 | Anti-ferroptosis | Kuang et al., 2021 |
| IRN | p53 | miR-122-5p/p53/SLC7A11 pathway | Anti-ferroptosis | Zhao et al.,2021 |
| FAC | Iron ions | a source of iron to induce iron overload | Promote ferroptosis | Zhao et al.,2021 |
