# Supplementary material for: Neuronal ferroptosis after intracerebral hemorrhage
Source: Front Mol Biosci. 2022 Aug 5;9:966478. doi: 10.3389/fmolb.2022.966478 (PMC9388724; doi:10.3389/fmolb.2022.966478)
Supplement: Supplementary file 2 [file DataSheet1.PDF]

Certificate of English Language Editing

Manuscript Title:

Neuronal Ferroptosis after Intracerebral Hemorrhage

Date of Revision:

June 6, 2022

Abstract:

Ferroptosis is a recently discovered type of cell death caused by the imbalance of intracellular redox metabolism. Ferroptosis is associated with various physiopathological processes, including normal development, ischemic organ damage, neurodegenerative diseases, and immune system activity, and plays a particularly important role in the occurrence and progression of intracerebral hemorrhage (ICH). Many ICH-induced regulators and signaling pathways of ferroptosis have been reported as promising targets for treating ICH. However, the mechanism of secondary neurological impairment after ICH remains unclear, and its relationship with neuronal ferroptosis caused by iron deposition after ICH remains to be elucidated in further research. In this article, we review the definition, characteristics, and inhibition methods of neuronal ferroptosis caused by iron deposition after ICH, and review the biomarkers for ferroptosis.

This document certifies that the manuscript listed above was copy edited for English language by LetPub, with regard to grammar, punctuation, spelling, and clarity. All of our language editors are native English speakers with long-term experience in editing scientific and technical manuscripts. We are committed to leveling the playing field for researchers whose native language is not English.

- Documents receiving this certification should be regarded as having undergone professional editorial revision for English language before submission. However, the authors may accept or reject LetPub's suggestions and changes at their own discretion and LetPub does not have editorial control over the submitted documents.
- The language quality of the submitted document is the sole responsibility of the submitting authors subject to those authors' adherence to LetPub's revisions and instruction. LetPub's provision of service does not constitute a guarantee or endorsement of the authors' work herein.
- Neither the research content nor the authors' intended meaning were altered in any way during the editing process.
- If you have any questions or concerns about this edited document, please contact us at support@letpub.com

LetPub
Your Manuscript Editor

LetPub is an author service brand owned and operated by Accdon LLC. Headquartered in the Boston area, we are a full-spectrum author services company with a large team of US-based certified language and scientific editors, ISO 17001 accredited translators, and professional scientific illustrators and animators. We advocate ethical publication practices and are an official member of the Committee on Publication Ethics (COPE).

For more information about our company, services, and partnership programs, please visit www.letpub.com.

© 2022 Accdon, LLC. All Rights Reserved. Tel: 1-781-202-9968 Email: info@accdon.com Address: 400 Fifth Ave, Suite 530, Waltham, MA 02451, United States
